# Practical Chemistry for Students and Physicians

**Published:** 1883-06

**Authors:** 


### PRACTICAL CHEMISTRY FOR STUDENTS AND PHYSICIANS.

This important field of study is divided into two parts, viz.: Theoretical chemistry and practical chemistry. The second, the more interesting, because the more useful part, can be properly understood, only, by a clear apprehension of the first. Chemistry deals with matter as such. But matter presents itself to the senses, not only as formless masses or disintegrated particles, like mountains or grains of sand, but in innumerable crystaline and organized forms.

Hence for the convenience of the student, all the objects of nature may be arranged respectively under the head of Physical Science and Natural Science.

Science.—Science is knowledge of nature classified and arranged.

Natural Science.—Natural science treats of the outer forms and inner structure of bodies; as a flower, a tree, a crystal. Hence botany, mineralogy and zoology are natural sciences.

Physicial Science.—Physical science considers only the matter of which those forms are constructed.

Chemistry.—Chemistry is a physical science, because it, too, treats of the properties of mere matter, whether hard or soft, light or heavy, combustible or non-combustible.

Science, therefore, may be divided for convenience into three great branches of study, viz.: Chemistry, Physics proper, and Natural Science.

Matter.—Matter is anything that effects our senses—anything that occupies space.

Mass, Molecule, Atom.—Matter is studied in three aspects; in the mass, as a world or a grain of sand; in the molecule, as a particle too minute to be reached by the senses, and in the atom, a particle of matter indefinably more minute than the molecule.

(TO BE CONTINUED.)

